# Lung cancer biopsy: Can diagnosis be changed after immunohistochemistry when the H&E-Based morphology corresponds to a specific tumor subtype?

**DOI:** 10.6061/clinics/2018/e361

**Published:** 2018-10-25

**Authors:** Fabiola Del Carlo Bernardi, Marcela Del Carlo Bernardi, Teresa Takagaki, Sheila Aparecida Coelho Siqueira, Marisa Dolhnikoff

**Affiliations:** I Departamento de Patologia, Faculdade de Medicina FMUSP, Universidade de Sao Paulo, Sao Paulo, SP, BR; II Graduacao em Medicina, Faculdade de Medicina FMUSP, Universidade de Sao Paulo, Sao Paulo, SP, BR; III Divisao Pulmonar, Instituto do Coracao (InCor), Hospital das Clinicas HCFMUSP, Faculdade de Medicina, Universidade de Sao Paulo, Sao Paulo, SP, BR

**Keywords:** Adenocarcinoma, Lung Cancer, p63, Squamous Cell Carcinoma, TTF1

## Abstract

**OBJECTIVES:**

Advancements in non-small cell lung cancer treatment based on targeted therapies have made the differentiation between adenocarcinoma and squamous cell carcinoma increasingly important. Pathologists are challenged to make the correct diagnosis in small specimens. We studied the accuracy of an immunohistochemical panel in subclassifying non-small cell lung cancer in routine small biopsies and compared the results with the diagnosis from resected lung specimens, autopsy samples or biopsied/resected metastases.

**METHODS:**

In total, 340 lung cancer biopsies were investigated for the expression of CK5, TTF1, p63 and surfactant.

**RESULTS:**

We characterized 166 adenocarcinomas and 124 squamous cell carcinomas. Overall, 85% of cases displayed binary staining (TTF1 positive/p63 negative, and vice versa). The diagnoses of ten cases with a morphology that indicated a specific tumor subtype were changed after immunohistochemistry (IHC). A second specimen was available for 71 patients, and the first diagnosis at biopsy was confirmed in 95% of these cases. Most non-small cell lung cancer cases present a binary immunohistochemical profile in small biopsies, contributing to good diagnostic accuracy with routine markers. In a small proportion of cases, the diagnosis can be changed after IHC even when the morphological aspects indicate one specific tumor subtype.

**CONCLUSIONS:**

We recommend that routine small biopsies of lung cancer without classic morphology should be subjected to a minimum immunohistochemical panel to differentiate adenocarcinoma from squamous cell carcinoma.

## INTRODUCTION

Major therapeutic advancements in lung cancer based on molecular testing have been observed over the last decade. Prior to the 2000s, lung cancer was classified into the following two major groups that received distinct treatments: small cell lung carcinoma (SCLC) and non-small cell lung carcinoma (NSCLC) including squamous cell carcinoma (SQCC), adenocarcinoma (ADC), large cell lung carcinoma (LCLC) and sarcomatoid carcinoma (SC).

Recent advancements in NSCLC treatment based on targeted therapies and evidence of drug cytotoxicity in specific histological subtypes have made the differentiation between ADC and SQCC increasingly important. For example, patients with SQCC treated with bevacizumab (anti-VEGF ligand antibody) may develop life-threatening hemorrhage, and treatment with pemetrexed (Alimta) may be ineffective 1. EGFR mutations and EML4-ALK rearrangements are almost exclusively observed in lung ADCs. Therefore, EGFR tyrosine kinase inhibitors are used as a first-line therapy in patients with advanced lung ADCs with EGFR mutations, and crizotinib has been used for advanced ADCs with ALK rearrangements 2.

Pathologists play a major role in personalized medicine for patients with lung cancer; however, the correct diagnosis of small specimens is challenging. The new WHO classification of lung tumors 3 proposes specific terminologies for ADC, SQCC and NSCLC in small biopsies or cytology based on both morphology and special staining and recommends that the diagnosis of NSCLC not otherwise specified (NSCLC-NOS) should be used as sparingly as possible. As most patients (80%) have advanced disease at the time of diagnosis, a small biopsy is frequently the only available material, and the morphology is often inadequate for the determination of the histological subtype 4. Immunohistochemistry (IHC) is currently the major tool to distinguish between ADC and SQCC in biopsies classified as NSCLC.

Few studies that evaluated the accuracy of IHC for classifying NSCLC have used actual biopsies, while most of the studies used tissue microarrays (TMAs) obtained from surgical specimens to mimic small samples 4-9. These studies have proposed an algorithm to separate ADC from SQCC, where cases positive for thyroid transcription factor (TTF)-1 were classified as ADC and cases for positive the p40/p63 protein were classified as SQCC. However, coexpression of p63 and TTF1 can occur, and it has been proposed that these cases should be classified as ADCs 10.

In the present study, we aimed to evaluate the accuracy of an immunohistochemical panel in subclassifying NSCLC in routine small biopsies and compare the results with resections of lung specimens, autopsy materials or metastasis resections when available.

## MATERIALS AND METHODS

We retrospectively examined consecutive small lung biopsies performed for lung cancer at our institute between 2010 and 2012. The biopsy samples were obtained from the files of the Hospital Pathology Division. All biopsies diagnosed as lung cancer via hematoxylin-eosin (H&E) staining were included in the study. The biopsies were classified as ADC, SQCC, NSCLC or poorly differentiated carcinoma based on their morphology.

For IHC, representative formalin-fixed, paraffin-embedded tissue blocks were cut into 4-µm thick sections. These sections were deparaffinized and subjected to antigen retrieval with sodium citrate buffer (pH 6.0) in a pressure cooker. Endogenous peroxidase was blocked using 5% hydrogen peroxide. Nonspecific staining was blocked in 2% normal swine serum. The slides were incubated with primary antibodies (anti-TTF1, clone 8G7G3/1, Zeta, dilution 1:500 (Thermo Fisher, Waltham, MA, USA); anti-surfactant, clone 32E12, Monsanto, dilution 1:2000 (St. Louis, MO, USA); anti-p63, clone 4A4, Zeta, dilution 1:8000 (Thermo Fisher, Waltham, MA, USA) and anti-cytokeratin (CK)-5, clone EP1601, Cell Marque, 1:20000 (Rocklin, CA, USA). and then reacted with a polymeric reagent combined with secondary antibodies and peroxidase (Reveal-Spring). Specific antigen-antibody reactions were visualized with 0.2% diaminobenzidine tetrahydrochloride and hydrogen peroxide. Counterstaining was performed using Mayer's hematoxylin. Normal lung tissue (TTF1 and surfactant) and amygdala (p63 and CK5) were used as positive controls. Immunostaining was considered positive when more than 1% of tumor cells were stained. Positivity was further defined as focal (up to 10% of tumor cells) or diffuse (more than 10% of tumor cells). In a subgroup of patients, the diagnosis in biopsy samples was compared with the diagnosis in lung resections, autopsy materials, metastasis resections or a second lung biopsy when available. Two pathologists (FDCB and MD) reviewed the cases and IHC staining.

### Ethics

The study was approved by the local ethics committee.

This work does not represent a clinical trial and was not registered as so.

## RESULTS

**Figure 1:**
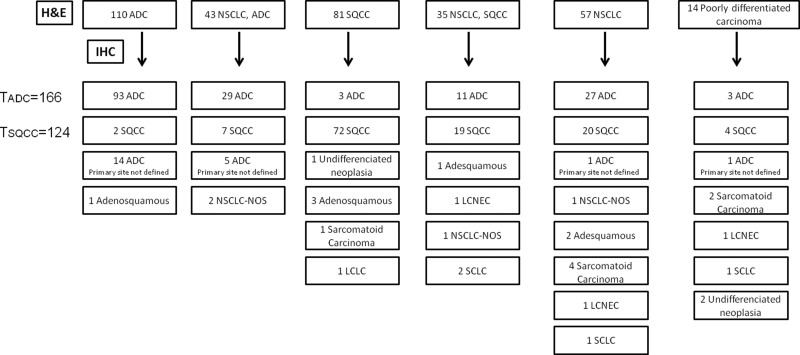
Distribution of diagnoses of 340 cases from H&E and IHC staining. ADC: adenocarcinoma; SQCC: squamous cell carcinoma; NSCLC: non-small cell lung cancer; NSCLC, ADC: NSCLC-probably ADC; NSCLC, SQCC: NSCLC-probably SQCC; LCLC: large cell lung carcinoma; LCNEC: large cell neuroendocrine carcinoma; NSCLC-NOS: NSCLC not otherwise specified; TADC: total cases of pulmonary adenocarcinoma; TSQCC: total cases of squamous cell carcinoma.

We analyzed 420 lung biopsies including 246 transthoracic core biopsies, 97 transbronchial biopsies, 64 endobronchial biopsies, 1 endobrochial and transbronchial biopsy, and 12 biopsies with no information regarding the sampling method. In all cases, there was a clinical suspicion of primary or metastatic cancer. After immunohistochemical staining, 80 (19.2%) cases were excluded (62 metastases and 18 with insufficient material). The remaining 340 cases (200 males and 140 females) of lung cancer were subclassified as shown in Figure 1.

Within the 166 pulmonary ADCs, 93 (56%) had morphological features readily recognized by H&E staining, 29 (17.5%) were diagnosed as NSCLC-probably ADC, 14 (8.4%) were diagnosed as SQCC or NSCLC-probably SQCC, and 30 (18.1%) did not display any obvious differentiating features (NSCLC or poorly differentiated carcinoma) (Figure 1 and Table 1).

**Figure 2:**
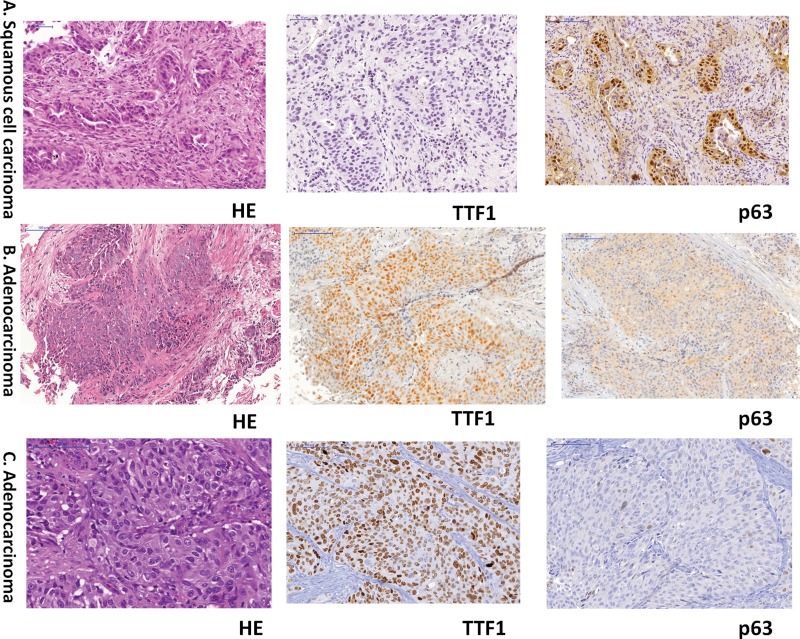
Representative photomicrographs of three cases for which diagnoses were changed after IHC. A: Lung cancer diagnosed as ADC by H&E staining, with a final diagnosis of SQCC after IHC (TTF1 and p63 staining). B and C: Lung cancer diagnosed as SQCC by H&E staining, with a final diagnosis of ADC after IHC (TTF1 and p63 staining).

In addition to these 166 cases, 3 biopsies were classified as ADC based on H&E staining, and their diagnoses were changed following IHC (Figures 1 and 2).

The diagnosis of pulmonary ADC (total of 166 cases) was determined when the biopsy showed focal or diffuse TTF1 positivity. Surfactant staining was used in 148 cases and was positive in 103 (69.6%). p63 protein and CK5 were focally positive in 30/148 (20.3%) and 22/141 cases (15.6%), respectively. The 14 pulmonary ADC cases that were first diagnosed as SQCC or NSCLC-probably SQCC based on H&E staining were diffusely positive for TTF1 and subsequently diagnosed as ADC; 8 of these were also positive for surfactant. In 3 cases first diagnosed with NSCLC based on H&E staining, both p63 and TTF1 were positive, but a final diagnosis of ADC was made because they were also positive for surfactant (Table 2).

Another 21 ADC cases had no confirmation of the primary site (Figure 1).

Among the 124 SQCC cases, 72 (58.1%) had a morphological aspect readily recognized by H&E staining, 19 (15.3%) were diagnosed as NSCLC-probably SQCC, 9 (7.3%) were diagnosed as ADC or NSCLC-probably ADC, and 24 (19.3%) did not display any obvious differentiating features (NSCLC or poorly differentiated carcinoma) (Figure 1 and Table 3).

In addition to these 124 cases, 7 biopsies were classified as SQCC based on H&E staining, and their diagnoses were changed following IHC (Figure 2). Therefore, a total of 10 cases with morphology that favored a specific tumor subtype had their diagnoses changed after IHC (3 ADC and 7 SQCC).

The p63 protein was positive in 119/124 (96%). The 5 SQCC cases that were negative for p63 were positive for CK5 and negative for TTF1. CK5 was positive in 116/119 cases (97.5%). TTF1 was focally positive in 5/121 (4.1%) cases that displayed morphologic features of SQCC and positivity for p63 and CK5. Three of these 5 cases had another sample available from biopsy or surgical resection, and 1 case had a final diagnosis of pleomorphic carcinoma, and 2 remained SQCC. Surfactant was negative in all cases of SQCC (Table 2).

Of the 290 cases classified as pulmonary ADC or SQCC, immunostaining for both TTF1 and p63 was performed in 269; 229 (85%) of these cases had binary staining, such as TTF1 positive/p63 negative, and vice versa.

The last group of NSCLC samples comprised 22 cases that presented as other histological subtypes as follows: 7 SCs, 7 adenosquamous carcinomas, 1 LCLCs, 3 large cell neuroendocrine carcinomas (LCNECs) and 4 NSCLCs-NOS. The immunohistochemical profiles of these cases is presented in Table 4.

In 71/340 (20.9%) cases (48 ADC, 19 SQCC and 4 other subtypes), a second specimen was obtained for comparison (second lung biopsy, resection of the primary tumor, biopsy/resection of a distant metastasis or autopsy material). Diagnostic agreement was observed in 95.7% of these cases. In 3 cases, the final diagnosis was changed from ADC to adenosquamous carcinoma, SQCC to SC, and adenosquamous carcinoma to SQCC.

## DISCUSSION

For many years, the pathological classification of lung cancer has been based on H&E-stained slides of surgical specimens. The 2015 edition of the WHO lung cancer classification recommends the use of IHC to differentiate ADC from SQCC, especially in biopsy specimens of NSCLC. This new recommendation is in accordance with many studies that describe immunohistochemical panels suitable for differentiating ADC from SQCC 3. However, most of these studies used TMAs to simulate a surgical biopsy and compared the IHC results of TMAs with the diagnosis based on surgical specimens to validate these panels 4. Few studies have compared actual lung biopsies with surgical specimens 9 because a large proportion of patients present with advanced-stage disease and do not undergo surgery.

In the present study, we showed that IHC was useful in differentiating pulmonary ADC from SQCC in small biopsies. The final diagnosis of these histological subtypes was determined in 85.3% of the total studied cases. As expected, most of the patients did not undergo surgical resection for histological confirmation. However, we were able to evaluate additional specimens from 71 cases, obtained from a second lung biopsy, resection of the primary tumor, biopsy/resection of a distant metastasis or autopsy. This second specimen was mainly available for the ADCs. Although a second specimen was available for only 21% of the patients, the first diagnosis was confirmed in 95% of these cases. This result validates the final diagnoses obtained after IHC in our series of biopsies.

In this study, we included not only cases classified as NSCLC but also biopsies with ADC or SQCC morphology. We demonstrated that in a small proportion of cases, IHC can alter the first diagnostic impression, indicating the value of performing IHC in all biopsy samples of pulmonary tumors.

Most of the ADC and SQCC cases (85%) had binary staining, such as TTF1 positive/p63 negative, and vice versa. The morphology observed by H&E staining indicated the correct diagnosis in 73% of the pulmonary ADC and SQCC cases and classified 6.5% of the cases as NSCLC. Previous reports demonstrated similar results, showing that 60-75% of pulmonary ADC and SQCC could be exclusively identified by morphology, and 7% of cases were classified as NSCLC 11,12. Our cases followed an international trend of a higher incidence of ADC (48.8% in our series) than of SQCC (36.7%) 13.

Eleven cases were classified as adenosquamous carcinoma (n=7) or NSCLC-NOS (n=4) when the IHC profile demonstrated double positivity or negativity, respectively. Surgical samples were available for 4 of these 11 cases, and only one final diagnosis was changed after resection (adenosquamous carcinoma to poorly differentiated SQCC). In 2015, the WHO recommended that cases with coexpression of p63 and TTF1 (either within the same cells or in different tumor cells) be classified as ADC when TTF1 and p63 are positive in the same tumor cells and as adenosquamous carcinoma when these markers are positive in different populations of tumor cells. The term NSCLC-NOS should be used when both markers are negative. Coexpression of p63 and TTF1 in tumors can be explained by the heterogeneity of lung cancer as demonstrated by Cadioli et al., who showed that one-third of their ADC samples expressed p63 or CK5 14. The frequency of positivity for squamous markers in ADCs varies according to the panel of markers. For example, p40 and desmocollin show higher specificity for SQCC and lower positivity in ADC 15,16. In the present study, we retrospectively examined consecutive small lung biopsies performed for lung cancer and used TTF1 and p63 as the main markers for ADC and SQCC, respectively. Although the use of p40 is currently being standardized at our institute, it has not been routinely used.

The WHO recommends that cases with sarcomatoid features should be classified as pleomorphic carcinoma only when the surgical specimen is available. In these cases, marked nuclear pleomorphism, malignant giant cells or spindle cell morphology should be present in at least 10% of the tumor cells. However, 7 of our cases presented marked sarcomatoid features at biopsy; among them, 4 were positive for TTF1 or p63, 3 were negative for both markers, and all of them were positive for both AE1/AE3 and vimentin. We believe that effort should be made to identify these cases even in biopsy samples for prospective studies regarding treatment and prognosis.

Twenty-one cases classified as ADC based on H&E staining (11% of ADC cases) lacked confirmation of the primary site. As these patients presented with a solitary pulmonary mass and did not show positivity for markers corresponding to other sites 10,11, these cases probably represent primary pulmonary tumors that are negative for both TTF1 and surfactant.

We conclude that most NSCLC cases present a binary immunohistochemical profile on small biopsies, contributing to good diagnostic accuracy with routine markers. In a small proportion of cases, the diagnosis may be changed after IHC even when the morphological aspects indicate a specific tumor subtype. Therefore, we recommend that routine small biopsies of lung cancer without classic morphology should be subjected to a minimum immunohistochemical panel (TTF1 and p63, p40 or CK5) to differentiate ADC from SQCC.

## AUTHOR CONTRIBUTIONS

Bernardi FDC was responsible for the conception and design of the study, participated in the analysis and interpretation of data and drafted the manuscript. Bernardi MDC, student of medicine, participated in the acquisition of clinical and pathological data. Takagaki T participated in the acquisition of clinical data. Siqueira SA performed the immunohistochemistry quality control and contributed to the data interpretation. Dolhnikoff M participated in the design of the study, data interpretation, drafted and revised the manuscript.

## Figures and Tables

**Table 1:** Distribution of the pulmonary adenocarcinomas when first classified by H&E staining.

H&E	ADC	NSCLC-probably ADC	SQCC or NSCLC-probably SQCC	NSCLC or poorly differentiated carcinoma	Total
	93 (56%)	29 (17.5%)	14 (8.4%)	30 (18.1%)	166

ADC: adenocarcinoma; SQCC: squamous cell carcinoma; NSCLC: non-small cell lung cancer.

**Table 2:** Positivity of the immunohistochemical markers for adenocarcinoma and squamous cell carcinoma.

IHC	TTF1	Surfactant	p63	CK5
ADC	166/166 (100%)	103/148 (69.6%)	30/148 (20.3%)	22/141 (15.6%)
SQCC	5/121 (4.1%)	0/114 (0%)	119/124 (96%)	116/119 (97.5%)

ADC: adenocarcinoma; SQCC: squamous cell carcinoma; IHC: immunohistochemistry; TTF: thyroid transcription factor; CK: cytokeratin.

**Table 3:** Distribution of squamous cell carcinoma when first classified by H&E staining.

H&E	SQCC	NSCLC-probably SQCC	ADC or NSCLC-probably ADC	NSCLC or poorly differentiated carcinoma	Total
	72 (58.1%)	19 (15.3%)	9 (7.3%)	24 (19.3%)	124

ADC: adenocarcinoma; SQCC: squamous cell carcinoma; NSCLC: non-small cell lung cancer.

**Table 4:** Immunohistochemical markers for histological subtypes other than adenocarcinoma and squamous cell carcinoma.

	TTF1	Surfactant	p63	CK5	AE1/AE3	Vimentin	Chromogranin
Sarcomatoid carcinoma (n=7)	2/7	1*/4	3/7	0/5	7/7	7/7	np
Adenosquamous carcinoma (n=7)	4/7	2*/7	7/7	6/7	np	np	np
Large cell lung carcinoma (n=1)	1/1	0/1	0/1	1/1	np	np	np
Large cell neuroendocrine carcinoma (n=3)	3/3	0/1	2*/3	1*/1	np	np	3/3
NSCLC-NOS (n=4)	0/4	0/3	0/4	0/3	4/4	np	np

*Focal; np: not performed.

TTF: thyroid transcription factor; CK: cytokeratin; AE1/AE3: pancytokeratin.
